# A one health indicator-based policy and implementation gap analysis of brucellosis control in Iran: A scoping review

**DOI:** 10.1371/journal.pntd.0014690

**Published:** 2026-11-06

**Authors:** Maryam Dadar, Zahra Baradaran-Seyed

**Affiliations:** Razi Vaccine and Serum Research Institute, Agricultural Research, Education and Extension Organization (AREEO), Karaj, Iran; Makerere University, UGANDA

## Abstract

**Background:**

Brucellosis remains endemic in Iran despite more than six decades of vaccination, surveillance, and test-and-slaughter activities. However, the national control program has not been systematically assessed against internationally recognized progressive-control frameworks. We conducted a descriptive and exploratory scoping review combined with an indicator-based policy and implementation gap analysis. The study was not designed to test a conventional epidemiological hypothesis or estimate causal effects.

**Methodology/Principal Findings:**

We developed 45 measurable indicators by adapting the Food and Agriculture Organization Stepwise Approach for the Progressive Control of Brucellosis across five domains: governance, surveillance, laboratory capacity, prevention and control, and communication. The indicators were informed by nine international normative sources and reviewed by three subject-matter experts. We searched PubMed, Scopus, Web of Science, and Google Scholar for scientific literature published between January 2000 and March 2026 and manually screened relevant publicly accessible official documents, including earlier legal and regulatory sources. Seventy-one eligible documents were included. Two reviewers independently scored all indicators using predefined criteria. Exact agreement was 86.7% (39/45), and weighted Cohen's kappa calculated from the original independent pre-consensus scores was 0.84 (95% CI 0.72–0.93). The six disagreements were resolved by consensus. Overall stage classification was determined separately using a rule-based algorithm based on documented program prerequisites rather than aggregated indicator scores. Under the adapted framework, Iran met the minimum documented prerequisites associated with Stage 3. Major implementation or documentation gaps remained in animal identification and traceability, early warning, laboratory partnerships, wildlife and nomadic-population risk management, joint risk assessment, and public reporting. Six interdependent policy priorities were identified.

**Conclusions/Significance:**

Iran has established important foundations for brucellosis control, but several integrated and elimination-oriented functions remain incomplete or undocumented. The Stage 3 classification is a framework-based interpretation of publicly available documentary evidence, not an official designation or an independently validated measure of national program performance. The framework may support systematic gap identification and program monitoring, but prospective and external validation is required before its broader application.

## Author summary

Brucellosis is an infection transmitted from animals to humans that causes chronic illness, livestock losses, and food-safety risks. Although Iran has implemented vaccination, testing, and public-education programs for more than six decades, the disease remains endemic. We developed 45 measurable indicators to examine the documented strengths and gaps of the national control program across governance, surveillance, laboratory capacity, prevention and control, and communication. We reviewed 71 scientific and publicly accessible official documents and assessed each indicator using predefined criteria. We found that Iran has established important foundations for brucellosis control and meets the minimum documented requirements associated with the third stage of the adapted progressive-control framework. However, several systems required for further progress remain incomplete or undocumented, including animal traceability, early warning, laboratory partnerships, wildlife and nomadic-population risk management, coordination between human and animal health authorities, and public reporting. We identified six interconnected policy priorities to address these gaps. Because our assessment relied on publicly available documents, failure to retrieve evidence does not necessarily mean an activity does not exist. The framework may support transparent planning and monitoring but requires further validation before broader use.

## Introduction

Brucellosis continues to pose a significant zoonotic threat in Iran, persisting despite six decades of mass vaccination, test‑and‑slaughter programs, and public education initiatives [1,2]. The National Brucellosis Control Program for sheep and goats has been implemented since 1963, yet the disease remains endemic [1]. Recent national data indicate that annual human incidence rates have not shown a sustained decline, fluctuating widely from 0.03 to over 200 cases per 100,000 population, with persistent hotspots identified in western and northwestern provinces [2]. Between 2011 and 2020, a total of 173,526 cases were reported across Iran, with an average incidence of 22 per 100,000 population, predominantly affecting males (58.2%) and rural residents (77%) [3]. The economic burden on the livestock sector is substantial, with annual losses exceeding US$200 million due to reproductive failures, decreased milk production, and trade restrictions [4]. The burden of human brucellosis, as measured by Disability‑Adjusted Life Years (DALYs), increased from 34.6 per 100,000 in 2009 to 71.4 per 100,000 in 2015 [5]. A systematic review and meta‑analysis of dairy products in Iran reported an overall *Brucella* spp. prevalence of 22% in dairy products, with the highest contamination in raw goat milk (27%) and traditional cheese (9%) [6].

Despite these ongoing challenges, Iran has yet to implement a fully integrated, stepwise control strategy that aligns animal, human, and environmental health efforts. Current interventions remain fragmented, characterized by uneven vaccination coverage—particularly in rural and nomadic populations—delayed financial compensation, inconsistent laboratory capacity, and limited multisectoral coordination [7].

Comparable challenges are observed in other endemic low‑ and middle‑income countries, where control efforts often fail to align with internationally recognized frameworks for progressive elimination [8]. The Food and Agriculture Organization (FAO) has introduced structured approaches, such as the FAO Stepwise Approach for the Progressive Control of Brucellosis (SAPCB) and the Staged Tool for the Elimination of Brucellosis (STEB) [9]. Nevertheless, few systematic evaluations operationalize these frameworks as measurable, actionable policy indicators at the national program level [10].

Iran serves as a pertinent example of this complexity. Despite maintaining a national brucellosis control program for several decades, which encompasses mass vaccination and passive surveillance [1,11], both human incidence and livestock prevalence remain elevated in various regions. Among nomadic populations in Iran, the prevalence of brucellosis has been reported as high as 6.3%, with virtually all cases linked to lifestyle factors including consumption of raw milk (94.7%), fresh cheese (100%), undercooked meat (95.1%), and animal skin contact (100%) [12]. Critically, progress toward internationally recognized elimination stages has not been systematically assessed using standardized indicators. Prior evaluations have largely been narrative, sector‑specific, or restricted to technical domains such as vaccine coverage [13]. A comprehensive, One Health‑oriented policy gap analysis is urgently needed to integrate governance, surveillance, laboratory capacity, prevention, and communication and guide strategic investment and reform [14].

Furthermore, the potential contribution of wildlife reservoirs—including recent molecular evidence of *Brucella* in snakes [15], rodents [16,17], and gazelles [18]—along with the role of dogs and nomadic lifestyle practices, has yet to be systematically addressed through a formal management plan [12,19,20]. To address this gap, we conducted a descriptive and exploratory scoping review combined with an indicator-based policy and implementation gap analysis. The study was not designed to test a conventional epidemiological hypothesis or estimate causal effects.

## Methods

### Study design and analytical framework

This study is a descriptive and exploratory scoping review combined with an indicator-based policy and implementation gap analysis. It was not designed to test a conventional epidemiological hypothesis, estimate causal effects, or generate population-level effect estimates. Conventional sample-size and statistical-power calculations were therefore not applicable because the unit of analysis was the document corpus rather than a human or animal study population. We conducted a comprehensive two-phase policy gap analysis. In Phase I, an indicator matrix was developed by adapting the FAO Stepwise Approach for the Progressive Control of Brucellosis (SAPCB; Stages 1–5) [9] across five domains: Governance, Surveillance, Laboratory, Prevention & Control, and Communication. These domains were also informed by the FAO Staged Tool for the Elimination of Brucellosis (STEB). In Phase II, evidence identified through the scoping review was mapped to the indicators and assessed using predefined scoring criteria.

**Table 1** presents the SAPCB stages as defined in the FAO 2025 guidance document, which describes five progressive stages from an unknown disease situation (Stage 1) through to self-declared freedom (Stage 5).

**Table 1:** Description of SAPCB stages and focus of the indicators used in this study (adapted from FAO, 2025).

SAPCB Stage	Description (FAO, 2025)	Focus of indicators in this study
Stage 1	Unknown disease situation with no structured control activities	Baseline assessment, initial seroprevalence survey, establishing a Veterinary Epidemiology Unit
Stage 2	The disease situation is understood, and a control program is planned.	National Multisectoral Brucellosis Committee (NMBC), NBCP drafted, economic evaluation.
Stage 3	Control program underway	Mass vaccination, laboratory strengthening, surveillance, reporting systems, compensation scheme
Stage 4	Brucellosis at low levels of occurrence or transmission	Targeted vaccination of young replacements, test-and-remove, early warning system, wildlife plan (if applicable)
Stage 5	Achieving and maintaining self-declared freedom from brucellosis (with or without vaccination)	WOAH-compliant testing (≥99.8% of herds free for 3 years), submission of dossier for self-declared freedom, cessation of vaccination (if free without vaccination)

### Phase I — Indicator development

#### Normative sources.

We selected several internationally recognized standards (Table 2) based on their global acceptance and direct relevance to brucellosis control, including WOAH Terrestrial Animal Health Code Chapter 8.4 [21], WOAH Manual of Diagnostic Tests Chapter 3.1.4 [22], WHO International Health Regulations Monitoring and Evaluation Framework [23], FAO Surveillance Evaluation Tool [24], FAO Laboratory Mapping Tool [25], CDC Brucellosis Reference Guide [26], and WOAH Performance of Veterinary Services Pathway [27]. Additional sources, including the Tripartite Guide and Joint Risk Assessment Operational Tool [28,29], were consulted to identify specific indicators. A rapid cross-check against a published One Health tool inventory [10] did not identify an additional major indicator domain requiring inclusion.

**Table 2:** Normative sources and tools used for indicator derivation.

Standard/Tool	Main Domain(s)	Key Application
CDC Brucellosis Reference Guide: Exposures, Testing, and Prevention [26]	Prevention & Control	Practical guidance for brucellosis control
FAO Laboratory Mapping Tool (LMT) — core and safety modules [25]	Laboratory	Assessing laboratory capacity
FAO Surveillance Evaluation Tool (SET) — core indicators [24]	Surveillance	Evaluating animal health surveillance systems
WHO International Health Regulations (2005) Monitoring and Evaluation Framework (IHR MEF), specifically SPAR and JEE technical areas [23]	Surveillance	Monitoring public health capacity
WOAH Manual of Diagnostic Tests and Vaccines for Terrestrial Animals, Chapter 3.1.4 (Brucellosis) [22]	Laboratory	Diagnostic and laboratory protocols
WOAH Performance of Veterinary Services (PVS) Pathway — Critical Competencies [27]	Governance	Evaluating veterinary services
WOAH Terrestrial Animal Health Code, Chapter 8.4 (Infection with *Brucella abortus*, *B. melitensis* and *B. suis*) [21]	Governance, Control	International animal health standards
FAO Stepwise Approach for the Progressive Control of Brucellosis (SAPCB) [9]	Governance, Control	Framework for progressive control and elimination
Tripartite Joint Risk Assessment Operational Tool [28,29]	Governance, One Health	Joint risk assessment protocol for zoonotic diseases

#### Indicator matrix.

The 45 indicators were developed by systematically translating the FAO SAPCB stage descriptors and key activities (Stages 1–5) into measurable, closed-ended questions across five operational domains: Governance, Surveillance, Laboratory, Prevention & Control, and Communication. The indicators used a standardized scoring scale: 0 = absent or not documented; 1 = partial or ad hoc; 2 = developed but incomplete; and 3 = fully operational and sustained. We mapped one or more candidate indicators to each relevant SAPCB requirement and cross-checked them against the nine normative sources listed in Table 2 to assess coverage of internationally recognized standards. This systematic cross-referencing process was informed by established health-policy and surveillance assessment frameworks [23,24]. We consolidated duplicate or substantially overlapping indicators and removed indicators that could not be assessed using publicly accessible Iranian scientific, official, or grey literature. To assess contextual relevance, clarity, and operational feasibility, the preliminary indicator matrix was independently reviewed by three subject-matter experts: a veterinary specialist with more than 15 years of experience in brucellosis surveillance, a veterinary clinical epidemiologist with 10 years of experience in One Health program evaluation, and a medical infectious disease specialist involved in refining the national brucellosis-control program. Each expert evaluated the indicators according to three qualitative criteria: (i) relevance to the burden and control context of brucellosis in Iran; (ii) clarity and measurability; and (iii) feasibility of evidence retrieval from Iranian scientific, official, and grey literature. The expert-review process was informed by established approaches to One Health tool mapping and health-policy evaluation [10,23]. Their feedback was used to refine the wording and scope of the indicators, after which all three experts agreed on the final set of 45 indicators. This process constituted conceptual adaptation and content review rather than formal psychometric, prospective, or external validation.

#### Stage classification algorithm.

To support transparent and reproducible stage assignment, we specified a study-specific, rule-based algorithm after evidence retrieval but before indicator scoring, and applied it after scoring was completed. Stage assignment was based on documented evidence for the specified program-level prerequisites and was not derived from a composite or mean indicator score. Any means presented in Fig 2 were calculated solely for descriptive visualization and were not used for stage classification. The resulting stage classification represents a framework-based interpretation of the available documentary evidence rather than an official FAO or WOAH designation or an independently validated measurement of national program performance.

The minimum documentary prerequisites used in the adapted rule-based algorithm were as follows:

- **Stage 1:** documented evidence of at least one national seroprevalence survey (S‑01) and existence of a multisectoral committee (G‑01) with formal terms of reference.
- **Stage 2:** documented evidence of an endorsed National Brucellosis Control Program (G-02) and a formal compensation system (G-04), regardless of its operational performance.
- Stage 3: documented evidence of routine vaccination in high-risk areas (PC‑02), at least one functional laboratory capacity (e.g., L-01 or L-04), and legal reporting obligations (G-03).
- **Stage 4:** documented evidence that brucellosis had reached low levels of occurrence or transmission, supported by molecular typing used in surveillance (S-05) and test-and-slaughter activities in low-prevalence populations or zones (PC-05).
- **Stage 5:** documented evidence of all five core indicators directly measurable from available documentation: a WOAH self-declaration dossier (G-06), three consecutive years of ≥99.8% herd freedom (S-07), WOAH reference laboratory designation (L-06), vaccination cessation per WOAH rules (PC‑07), and public annual reporting (C-06). Additional WOAH criteria (e.g., absence of clinical cases, movement controls) are treated as confirmatory requirements for formal dossier submission and are not operationalized as separate prerequisites.

For country-level stage classification, S-01 was considered present when a national seroprevalence survey was documented, and PC-02 was considered present when routine vaccination in high-risk areas was documented. The 10-year recency criterion for S-01 and the ≥ 80% coverage criterion for PC-02 were performance benchmarks for assigning an indicator score of 3 and were not treated as independent requirements for attaining Stage 1 or Stage 3, respectively.

It is essential to distinguish between two related but distinct concepts in this framework:

- **Adapted SAPCB Stage:** a country-level classification that reflects the overall maturity of the national brucellosis control program, based on whether the specified prerequisites of that stage and all preceding stages have been met.
- **Indicator Score:** an indicator-level measure on a scale from 0 to 3 reflecting the documented operational status of a specific policy, system, or capacity, irrespective of the country's overall stage classification.

Classification at Stage 3 therefore indicates that the minimum documentary prerequisites specified for progression through Stage 3 were met. It does not imply that every operational capacity associated with Stage 3 or subsequent stages was fully implemented. Consequently, a Stage 3 classification can coexist with low or zero scores for individual indicators representing incomplete, undocumented, or more advanced program functions.

Tables 3–7 present the individual indicator scores. Fig 2 presents domain-by-stage mean scores solely as a descriptive visualization; these means were not used to determine the overall stage classification. We determined the overall stage separately using the rule-based algorithm described above. The complete indicator matrix is provided in Table B in S1 Appendix; the complete reference list is provided in Table C in S1 Appendix; the indicator-level scoring justifications are provided in Tables D–H in S1 Appendix; and the stage-classification summary is provided in Table I in S1 Appendix.

### Phase II — Scoping review of Iranian evidence

#### Search strategy.

We searched PubMed, Scopus, Web of Science, and Google Scholar for literature published from January 2000 to March 2026. Official legal and regulatory documents identified through manual screening were eligible regardless of publication date, as long as they remained directly relevant to the national brucellosis-control framework. Search terms combined four domains: disease (brucellosis, *Brucella*, Malta fever), control (policy, surveillance, vaccination, test‑and‑slaughter, SAPCB), geography (Iran), and One Health (zoonotic, multisectoral). No language restriction was applied initially. The full search syntax for each database is provided in Table A in S1 Appendix. We manually screened reference lists of included articles and key official documents (e.g., IVO and MOHME executive instructions).

#### Eligibility criteria.

We included original research, systematic reviews, policy analyses, program evaluations, technical reports, and official regulations/documents that addressed brucellosis prevention, control, surveillance, or elimination in Iran with clear policy relevance. We excluded purely clinical studies without programmatic linkage, laboratory-characterization studies without direct relevance to one or more of the predefined indicators, single-case reports, and documents without full text. This scoping review approach followed established guidelines for policy-relevant systematic reviews [30,31]. The final evidence base (document corpus) comprised 71 documents meeting the eligibility criteria.

#### Screening and data extraction.

Two reviewers (ZBS and MD) independently screened titles and abstracts and subsequently assessed the full texts. Disagreements were resolved by consensus. From each included document, evidence relevant to one or more of the 45 indicators was extracted, including information concerning laws, program coverage, surveillance functions, and laboratory capacity. No sample-size or statistical-power calculation was performed, as the study was not designed for hypothesis testing and the unit of analysis was the document corpus.

### Scoring and gap analysis

Two reviewers independently scored all 45 indicators using the predefined indicator-specific criteria. Exact agreement was observed for 39 of the 45 indicators, corresponding to a raw agreement rate of 86.7%. For the remaining six indicators, the independent scores differed by one category on the 0–3 ordinal scale. We calculated weighted Cohen's kappa with quadratic weights using the original independent pre-consensus scores (κ = 0.84, 95% CI 0.72–0.93). The six disagreements were subsequently resolved through discussion and consensus after both reviewers re-examined the supporting evidence.

The final indicator scores were assessed and reported individually and were not combined into a composite score or used to determine the overall SAPCB stage. Domain-by-stage means were calculated only for descriptive visualization in Fig 2 and were not used for stage classification. Indicator scores reflect the application of the predefined criteria to the available documentary evidence and are distinct from the information reported directly in the source documents.

Following the initial scoring and consensus process, an independent third reviewer identified additional corroborating evidence relevant to four indicators. The two original reviewers reassessed these indicators in light of the additional evidence and agreed on the final scores. These subsequent evidence-based revisions were not included in the inter-rater reliability calculation.

Scores of 0 or 1 were classified as gaps, and scores of 0 were considered critical. The analysis of the gap patterns informed the identification of six evidence-informed policy priorities for brucellosis control in Iran, according to the established gap analysis frameworks for health systems [23,24].

### Ethical statement

This study exclusively utilized published literature and publicly accessible official documents. No primary data involving humans or animals were collected; the unit of analysis was the document corpus. Therefore, ethics approval was not required in accordance with standard guidelines for secondary research [32].

## Results

### Retrieved documents

The scoping review yielded 506 records from four major databases. After removing 217 duplicates, 289 titles and abstracts were screened for eligibility. Full texts of 186 records were assessed, and 71 met the inclusion criteria. The selection process is detailed in the PRISMA flow diagram (Fig 1). Normative sources used for indicator development were considered separately from the 71-document evidence corpus and are listed in Table 2.

**Fig 1:**
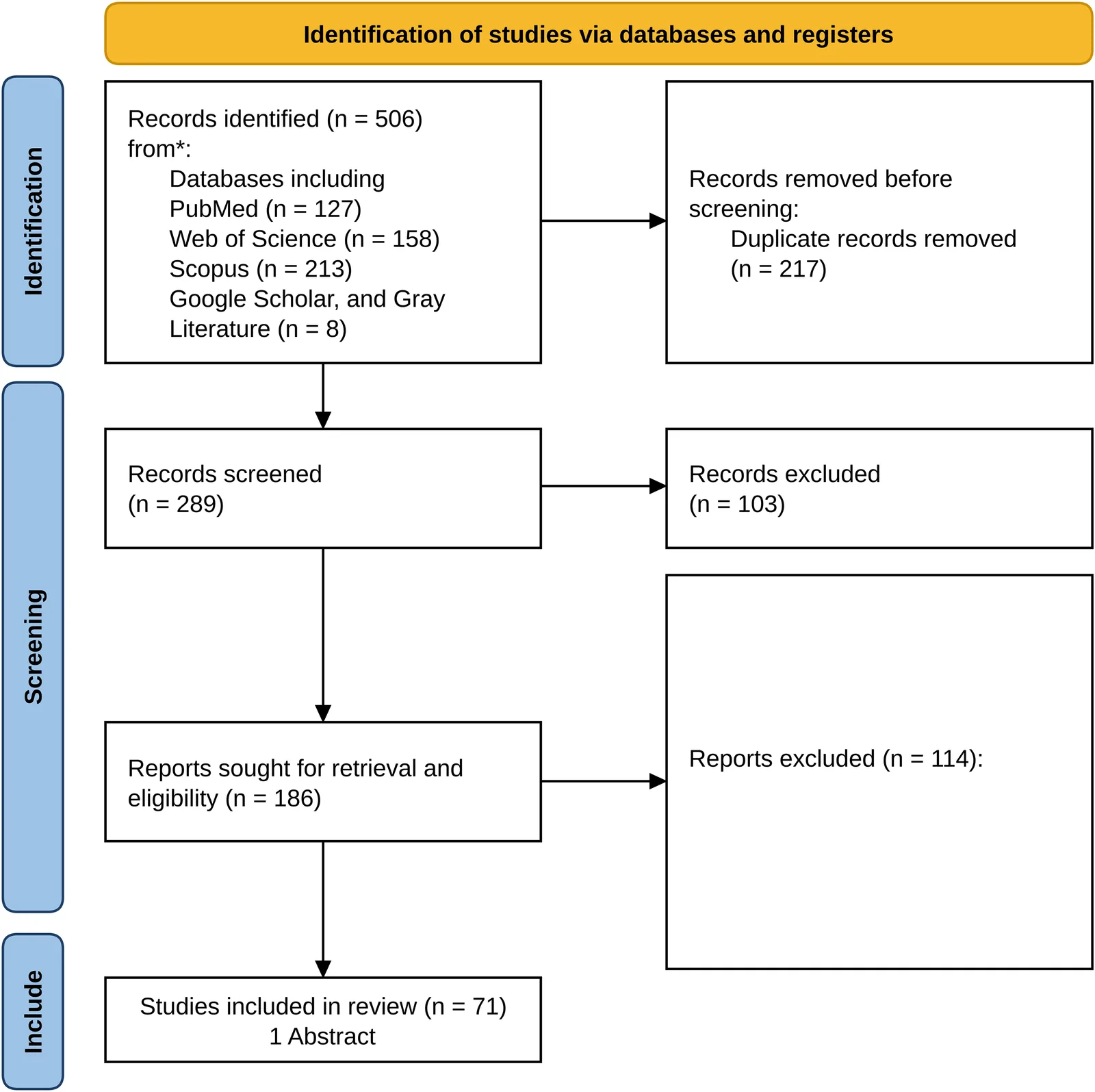
PRISMA 2020 flow diagram for policy gap analysis of brucellosis control in Iran.

### Analytical framework and 45 indicators

The analytical framework integrated the FAO SAPCB stages (1–5) with five key domains: Governance, Surveillance, Laboratory, Prevention & Control, and Communication, drawing on nine international normative sources [9,21–29]. The full matrix of 45 measurable indicators, each with predefined scoring criteria on a scale from 0 to 3, is provided in Table B in S1 Appendix. The study distinguished documentary findings reported by the included sources from the indicator scores and stage interpretation generated by applying the study's criteria.

Applying the adapted rule-based algorithm described in the Methods, Iran met the minimum documented prerequisites associated with SAPCB Stage 3. This classification represents a framework-based interpretation of the available documentary evidence rather than an official SAPCB designation or an independently validated measurement of national program performance. The classification was supported by documented evidence of a national seroprevalence survey (S-01, score 3), a multisectoral committee (G-01, score 2), a published national control program (G-02, score 2), legal reporting obligations (G-03, score 2), a formal but operationally incomplete compensation system (G-04, score 2), routine vaccination in high-risk areas (PC-02, score 2), and functional laboratory capacity (L-01, score 2; L-04, score 3). Several operational indicators associated with advanced control and elimination remained incomplete or undocumented. We interpreted individual indicator scores and overall stage classification separately, as described in the Methods.

Molecular typing used in surveillance (S-05, score 2) and limited test-and-slaughter activities (PC-05, score 1) represented components of transition towards Stage 4. However, the available documentary evidence did not establish that brucellosis had reached low levels of occurrence or transmission. Stage 4 was therefore not assigned under the adapted algorithm.

The distribution of scores across domains is summarised in Tables 3–7. Key findings for each domain are highlighted below (Fig 2).

**Fig 2:**
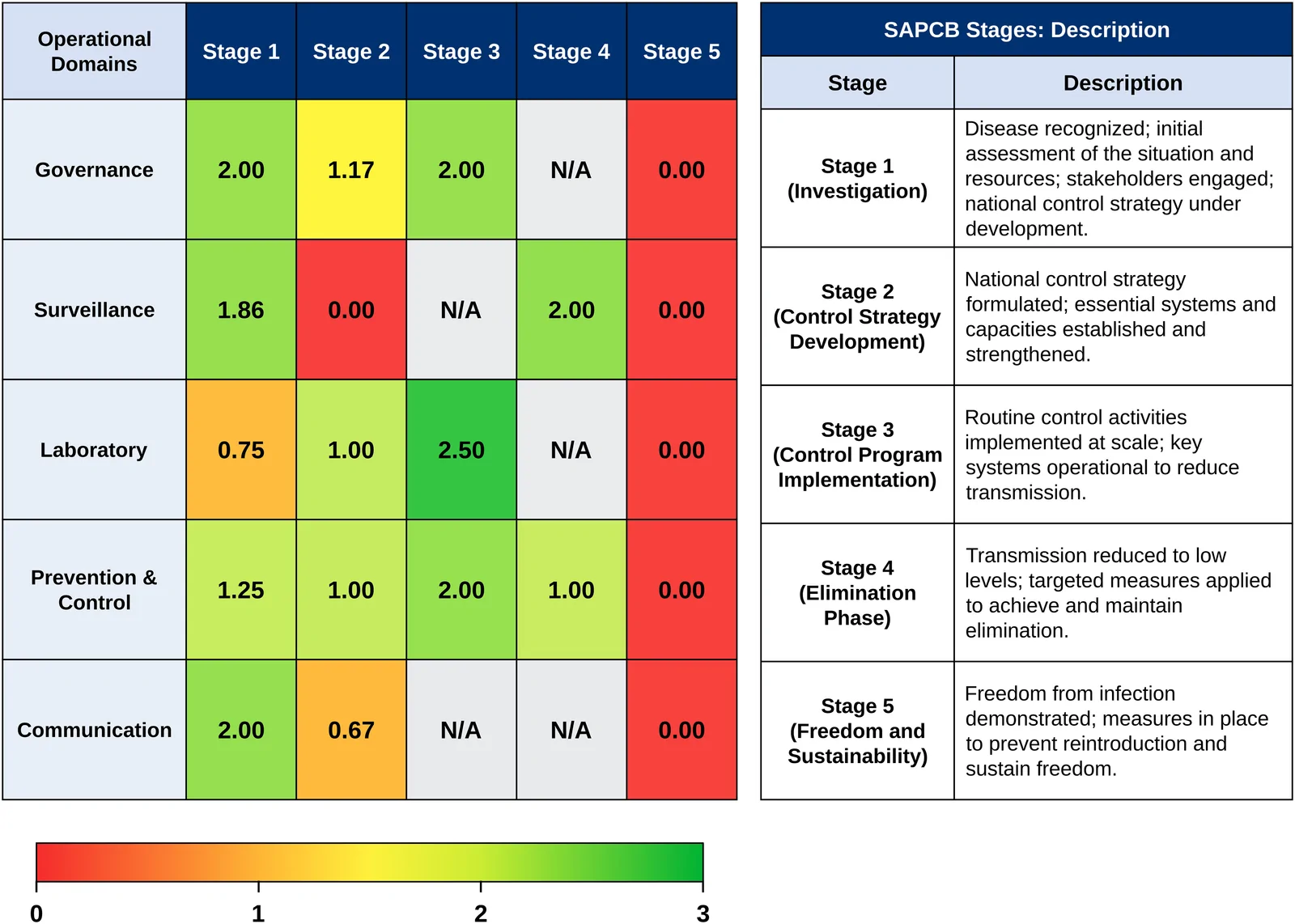
Heatmap illustrating mean indicator scores across the five operational domains and the adapted SAPCB stages. The color gradient represents mean indicator scores ranging from 0 to 3. Stage 3 values are based on the documented program prerequisites specified in the rule-based algorithm: legal reporting obligations (G-03), routine vaccination in high-risk areas (PC-02), and functional laboratory capacity (L-01 and L-04). Stage 4 values are based on molecular typing used in surveillance (S-05) and limited test-and-slaughter activities (PC-05). Stage 5 values are based on the five core documentary indicators specified in the algorithm (G-06, S-07, L-06, PC-07, and C-06; all score 0). Domain–stage cells without assigned indicators are shown as not applicable (N/A) and were excluded from mean calculations. The heatmap is descriptive; overall country-level stage classification was determined separately using the rule-based algorithm described in the Methods.

#### Governance (G01–G09).

Iran possesses a formal multisectoral committee (score 2) and a published National Brucellosis Control Program (score 2). While legal requirements for abortion reporting and a compensation system exist, enforcement is incomplete (both score 2). The compensation system has been a persistent challenge; over 60 years of program implementation, delayed payments and inadequate compensation (covering only 75% of animal value) have reduced farmer compliance [1,33]. Notable gaps scoring 0 or 1 included no publicly accessible evidence of a formal plan for transition from mass to targeted vaccination (G-05, score 0) or a WOAH self-declaration dossier (G-06, score 0), incomplete animal identification and traceability—particularly in rural and nomadic systems (G-08, score 1)—and no publicly accessible evidence of a formal joint human–animal risk-assessment protocol (G-09, score 0) (**Table 3**).

**Table 3:** Governance domain indicators, indicator stage in the adapted matrix, score, and key supporting sources.

ID	Indicator	Indicator stage in the adapted matrix	Score	Key supporting sources
G-01	Multisectoral committee	1	2	[13,34,35]
G-02	National Brucellosis Control Program (NBCP)	2	2	[1,36,37]
G-03	Legal obligation to report abortions	3	2	[38,39]
G-04	Compensation system	2	2	[1,33,34,39]
G-05	Plan for transition from mass to targeted vaccination	2	0	No publicly accessible evidence identified
G-06	WOAH self‑declared freedom dossier	5	0	No publicly accessible evidence identified
G-07	Full‑time official veterinarian per county	2	2	[38,39]
G-08	Animal identification/traceability system	2	1	[1,39]
G-09	Joint risk assessment protocol (human-animal)	2	0	No publicly accessible evidence identified

Scores (0–3) reflect the operational status of each indicator based on the available documentary evidence. They are distinct from the overall SAPCB stage classification.

The stage shown for each indicator denotes its placement within the adapted indicator matrix and is distinct from the rule-based algorithm used to determine the overall country-level stage classification.

#### Surveillance (S01–S12).

Strengths in surveillance include a national seroprevalence survey conducted within the past decade (S01, score 3) and established molecular typing capabilities (S05, score 2). However, there are significant deficiencies: the absence of an early warning system (S06, score 0), no publicly accessible evidence of three consecutive years of ≥99.8% herd freedom (S07, score 0), missing abortion-to-confirmation metrics (S10–S11, score 0), and insufficient systematic data sharing between human and animal health sectors (S04, score 1) (**Table 4**). Iran has a detailed written protocol for abortion monitoring with defined time intervals (S-03). Its reported nationwide implementation across industrial, semi-industrial, and traditional farms, together with mandatory testing in villages reporting human brucellosis cases, supported a score of 3. Studies have reported delays in human diagnosis, with mean times from symptom onset to diagnosis of 4.02–6.56 months in some provinces, highlighting limitations in diagnostic timeliness rather than providing a direct measure of abortion-to-laboratory confirmation [40]. Trend analyses in Mazandaran province (2006–2016) showed a 15% overall decrease in incidence, with a 66.2% decrease between 2006 and 2008 followed by a 7% increase thereafter, highlighting the fluctuating nature of control efforts [41].

**Table 4:** Surveillance indicators for brucellosis in Iran (S01–S12).

ID	Indicator	Indicator stage in the adapted matrix	Score	Key supporting sources
S‑01	National seroprevalence survey (last 10 years)	1	3	[1,42]
S‑02	Passive human surveillance with case definition	1	3	[3,36,37]
S‑03	Abortion monitoring system	1	3	[1,33,39]
S‑04	Data sharing (human-animal health)	1	1	[37,40,43]
S‑05	Molecular typing (MLVA, cgMLST) in surveillance	4	2	[44–46]
S‑06	Early warning system	2	0	[39]
S‑07	Three years of ≥99.8% herd freedom	5	0	No publicly accessible evidence identified
S‑08	% of counties regularly reporting livestock brucellosis	1	1	[39]
S‑09	% of epidemiological units screened per year	1	1	[39]
S‑10	Median time from abortion → lab confirmation	2	0	[39,40]
S‑11	% of abortion samples receiving a lab result	2	0	No publicly accessible evidence identified
S‑12	WHO case classification for humans	1	1	[36]

Scores (0–3) reflect the operational status of each indicator based on the available documentary evidence. They are distinct from the overall SAPCB stage classification.

#### Laboratory (L01–L09).

The national reference laboratory demonstrates the capacity to differentiate vaccine and field strains using PCR (L04, score 3). Culture capacity was documented, while BSL-3 capacity was not confirmed in the reviewed evidence (L02, score 1). According to WOAH standards [22], the recommended serological tests for brucellosis diagnosis include RBT, CFT, and ELISA, with PCR recommended as a complementary method. However, important deficiencies remain: no formal twinning with a WOAH/FAO reference laboratory was documented (L05, score 0), no WOAH reference laboratory designation was identified (L06, score 0), and no publicly accessible written waste-disposal protocol was identified (L08, score 0) (**Table 5**).

**Table 5:** Laboratory capacity indicators for brucellosis diagnosis, strain differentiation, and quality systems in Iran (L01–L09).

ID	Indicator	Indicator stage in the adapted matrix	Score	Key supporting sources
L‑01	National lab with RBT and limited ELISA (no CFT)	3	2	IVO labs (RBPT, SAT, i‑ELISA available); No CFT Test; WOAH recommends CFT as a confirmatory test [22,47,48]
L‑02	BSL‑3 + culture capacity	1	1	BSL-3 capacity not confirmed in the reviewed evidence; [49] (WGS research-based, culture); [39]— Razi Institute; [44]
L‑03	Quality management system (ISO)	1	0	Not documented in the retrieved evidence
L‑04	Differentiation of vaccine vs field strains by PCR	3	3	[39] — PCR possible from aborted fetus, milk, uterine discharges [48]; consistent with WOAH recommendations [22]
L‑05	Twinning with WOAH/FAO reference lab	2	0	[50] (database comparison); no formal twinning
L‑06	WOAH reference laboratory designation	5	0	No publicly accessible evidence identified
L‑07	Access to selective media (Farrell/CITA)	1	2	[46,49]
L‑08	Written waste disposal protocol	1	0	No publicly accessible protocol identified
L‑09	Number of *Brucella* isolates per year	2	2	6-60/year [46,49]

*Scores (0–3) reflect the operational status of each indicator based on the available documentary evidence. They are distinct from the overall SAPCB stage classification.*

#### Prevention & Control (PC01–PC08; Table 6).

A national control strategy is in place (PC01, score 2), and routine vaccination is implemented in high-risk areas, though coverage remains below target levels (PC02, score 2). Over the program's 60-year history, vaccination coverage has fluctuated significantly, with challenges in reaching nomadic and rural populations [1]. Major gaps include incomplete planning for wildlife and nomadic-population risks (PC-06, score 1) and no documented formal transition plan for vaccination cessation or restriction under the applicable WOAH requirements (PC-07, score 0). Test-and-slaughter activities in low-prevalence settings were documented only on a limited basis, principally on industrial farms (PC05, score 1), while testing and slaughter programs covered less than 1% of the sheep and goat population [1] (Table 6).

**Table 6:** Prevention and control indicators for brucellosis in Iran (PC01–PC08).

ID	Indicator	Indicator stage in the adapted matrix	Score	Key supporting sources
PC‑01	National control strategy	1	2	[1,51]
PC‑02	Routine vaccination in high-risk areas (≥80% coverage)	3	2	[1,39,42,52]
PC‑03	Post‑vaccination serological monitoring	1	0	[48]
PC‑04	Program to promote pasteurization	1	1	[6,39,53,54]
PC‑05	Test‑and‑slaughter in low-prevalence zones	4	1	[1,48,55]
PC‑06	Wildlife brucellosis/nomadic lifestyle plan	2	1	[12,56]
PC‑07	Vaccination stopped according to WOAH rules	5	0	No publicly accessible evidence identified
PC‑08	Standard treatment available at the primary level	1	2	[36,57]

Scores (0–3) reflect the operational status of each indicator based on the available documentary evidence. They are distinct from the overall SAPCB stage classification.

#### Communication (C01–C07).

A national KAP survey has not been documented, although several regional surveys are available (C01, score 2). Documented national risk-communication activities and planning elements remain incompletely implemented (C02, score 2). Joint human-animal risk communication following outbreaks occurs sporadically (C04, score 1), and no publicly accessible annual reporting was identified (C06, score 0) (**Table 7**). Studies consistently show moderate knowledge levels but low practice scores among livestock breeders, with education level being a significant predictor of knowledge and attitudes [53,58]. Among nomadic populations, knowledge of transmission routes is particularly low, contributing to high-risk practices [12].

**Table 7:** Communication indicators for brucellosis in Iran (C01–C07).

ID	Indicator	Indicator stage in the adapted matrix	Score	Key supporting sources
C‑01	National KAP survey	1	2	[53,58,59]
C‑02	National risk communication strategy	1	2	[39]
C‑03	Educational materials in local languages	1	2	[60,61]
C‑04	Joint (human-animal) risk communication after outbreaks	2	1	[13]
C‑05	Feedback mechanism for livestock owners	2	1	[62]
C‑06	Public annual reports on brucellosis status	5	0	No publicly accessible evidence identified
C‑07	Joint Risk Assessment (JRA) results translated into local messages	2	0	No publicly accessible evidence identified

Scores (0–3) reflect the operational status of each indicator based on the available documentary evidence. They are distinct from the overall SAPCB stage classification.

### Critical gaps (indicators scoring 0 or 1)

Indicators scoring 0 or 1 were further classified into three gap types adapted from the One Health literature [14,28]: Type I (knowledge gap) --- no retrievable evidence; Type II (implementation gap) --- policy or guideline exists, but execution is incomplete; and Type III (structural gap) --- no national policy, legal mandate, or formal institutional mechanism exists. This typology distinguishes between gaps that require research investment, operational strengthening, or legislative action.

The most notable deficiencies include no publicly accessible evidence of an early warning system (S06), no documented formal laboratory-twinning arrangement (L05), incomplete planning for wildlife and nomadic-population risks (PC06), and no publicly accessible annual reporting (C06).

Table 8 presents selected high-priority gaps relevant to current program strengthening and progression beyond Stage 3. It is not intended as an exhaustive list of all indicators scoring 0 or 1. We assigned a primary gap type to each indicator based on the dominant limitation documented in the reviewed evidence.

**Table 8:** Selected priority gaps among indicators scored 0 or 1 across the five operational domains of brucellosis control in Iran.

Domain	Indicator	Score	Primary Gap Type	Specific gap
**Governance**	G-05	0	Type I	No publicly accessible evidence of a formal plan for transition from mass to targeted vaccination was identified
	G-06	0	Type I	No publicly accessible WOAH self-declaration dossier or documentation of dossier preparation was identified
	G-08	1	Type II	Animal identification and traceability were partially implemented, with substantial gaps in rural and nomadic livestock systems
	G-09	0	Type I	No publicly accessible formal joint human–animal risk-assessment protocol was identified
**Surveillance**	S-06	0	Type III	No dedicated operational early-warning system for brucellosis was documented
	S-07	0	Type I	No publicly accessible documentation of three consecutive years with ≥99.8% of herds free from brucellosis was identified
	S-10	0	Type I	No national abortion-to-laboratory-confirmation performance metric was identified
	S-11	0	Type I	No publicly accessible national data on the proportion of abortion samples receiving laboratory results were identified
**Laboratory**	L-05	0	Type III	Informal international collaboration was documented, but no formal WOAH/FAO laboratory-twinning mechanism was identified
	L-06	0	Type I	No publicly accessible evidence of WOAH brucellosis reference laboratory designation was identified
	L-08	0	Type I	No publicly accessible written protocol for *Brucella*-contaminated waste disposal was identified
**Prevention & Control**	PC-03	0	Type I	No publicly accessible evidence of systematic post-vaccination serological monitoring was identified
	PC-06	1	Type II	Preliminary recognition and limited activities addressing wildlife or nomadic-population risks were documented, but no comprehensive national management plan was identified
	PC-07	0	Type III	No formal decision or documented transition mechanism for vaccination cessation or restriction under the applicable WOAH requirements was identified
**Communication**	C-04	1	Type II	Joint human–animal risk communication was documented, but implementation was occasional and not systematic
	C-06	0	Type I	No publicly accessible annual national report on brucellosis status was identified
	C-07	0	Type I	No publicly accessible evidence of translating formal joint risk-assessment findings into locally tailored communication messages was identified

### Priority evidence-informed recommendations

Based on the gap analysis, six evidence-informed priorities for advancing brucellosis control in Iran were identified:

I **Nationwide animal identification and traceability (addresses G08)** — prerequisite for movement control, particularly for rural and nomadic livestock systems.
II **Compensation system reform (G04)** — faster payments and insurance linkage; essential for farmer compliance with test-and-slaughter programs.
III **Early warning system development (S06)** — using syndromic abortion surveillance and molecular typing; would help address the 4–6-month diagnostic delays observed in some provinces [40].
IV **Formal laboratory twinning (L05)** — with a WOAH/FAO reference laboratory to strengthen diagnostic capacity, including CFT capability as recommended by WOAH standards [22].
V **Institutionalized One Health governance (G01, G09)** — legally mandated joint risk assessment to address fragmented coordination.
VI **Sustained national awareness campaign with annual public reporting (C02, C06)** — leveraging findings from KAP studies showing education as a key predictor of knowledge and attitudes [53,58]; must address high-risk nomadic lifestyle practices [12] and promote pasteurization given 22% dairy contamination [6].

These recommended actions are interdependent and together establish a strategic roadmap for transitioning from SAPCB Stage 3 to Stages 4 and 5.

## Discussion

Despite extensive vaccination and test-and-slaughter activities over several decades, brucellosis remains endemic in Iran. Using an adapted 45-indicator framework informed by the FAO SAPCB stages and related international standards, this study provides a literature-based, stage-specific assessment of documented policy and implementation gaps in a high-burden setting. Its main contributions are: (i) development of a transparent indicator matrix linking international standards to national policy and program functions; (ii) a framework-based assessment showing that Iran has established foundational capacities associated with Stage 3, while several functions required for advanced control and elimination remain incomplete or undocumented; and (iii) identification of six policy priorities for further program development. This approach is consistent with previous One Health policy evaluations in endemic settings [8,14].

### Documentary evidence is consistent with the minimum prerequisites associated with Stage 3

Under the adapted rule-based algorithm, Iran met the minimum documented prerequisites associated with Stage 3. The supporting evidence included a national seroprevalence survey (S-01, score 3), a multisectoral committee (G-01, score 2), a documented national control program (G-02, score 2), legal reporting obligations (G-03, score 2), a formal but operationally incomplete compensation system (G-04, score 2), routine vaccination in high-risk areas (PC-02, score 2), and functional laboratory capacity (L-01, score 2; L-04, score 3). This classification represents a framework-based interpretation of the available documentary evidence rather than an official SAPCB designation or an independently validated measurement of national program performance. Similar challenges in progressing from foundational control capacities towards elimination have been described in other endemic settings [8,63].

Crucially, stage classification must be distinguished from indicator scoring. Stage classification reflects whether the specified minimum documentary prerequisites were met, whereas indicator scores describe the documented operational status of individual program functions. Several indicators associated with advanced control and elimination remained incomplete or undocumented, including some that scored 0. The identified gaps may constrain program implementation; however, the available evidence demonstrates policy and implementation gaps rather than direct causal effects. The coexistence of a Stage 3 classification with low or zero individual indicator scores is therefore not necessarily contradictory, because the two measures represent different levels of assessment.

A notable asymmetry emerged in the laboratory domain. Functional laboratory capacities assigned to Stage 3 were relatively strong (mean score 2.50, based on L-01 and L-04), whereas foundational Stage 1 infrastructure remained weaker (mean score 0.75, based on L-02, L-03, L-07, and L-08), with gaps in quality management, confirmed BSL-3 capacity, and written waste-disposal procedures. More broadly, the most critical zero- or low-scoring gaps clustered around four areas: no publicly accessible evidence of an early warning system (S-06), lack of formal WOAH/FAO reference laboratory twinning and designation (L-05 and L-06), incomplete planning for wildlife or nomadic-population risks (PC-06), and no publicly accessible annual reporting (C-06).

Collectively, these findings indicate that Iran has established the “hardware” of control—vaccination, basic surveillance, and diagnostic capacity—but still lacks several elements of the “software” required for elimination, including integration, real-time data use, international laboratory partnerships, and community engagement. This pattern has also been recognized as a challenge in other endemic and middle-income settings [8,63]. Evidence from Iran's long-running national program supports this interpretation: vaccination coverage has rarely reached the 80% threshold, and test-and-slaughter activities have covered less than 1% of the sheep and goat population [1].

### Comparison with other countries and structural determinants

Direct international comparisons are limited, but experiences from Georgia, Mongolia, Kyrgyzstan, and Tunisia—countries implementing SAPCB-inspired programs—show a similar trajectory: initial reductions in prevalence through mass vaccination, followed by stagnation during the transition to targeted vaccination and test-and-slaughter strategies [9,64]. In contrast, countries that achieved documented freedom from brucellosis (such as several Western European nations) prioritized early investments in animal identification, twinning of reference laboratories, and enduring public–private partnerships [65]. The gaps identified in Iran thus mirror well-documented barriers to eradication [63,65]. Our framework is not a substitute for the official FAO STEB (Staged Tool for the Elimination of Brucellosis). STEB is a self‑assessment instrument intended for national veterinary services to conduct internal evaluations. In contrast, our approach is an evidence-reconstruction method based on publicly available literature and official documents that provides a framework-based program profile. The two approaches are complementary, and our findings can inform the official STEB self‑assessment process.

The persistence of Stage 4–5 gaps reflects deeper structural determinants, which can be grouped into four interconnected challenges:

**Fragmented governance:** The multisectoral committee established in the mid-1990s lacks independent budgetary authority and convenes irregularly, potentially hindering effective joint risk assessment (G09, score 0) and systematic data sharing (S04, score 1) [7,35].
**Incomplete incentive structure:** Compensation (G04, score 2) currently covers only 75% of animal value and is subject to delays, which may reduce farmer compliance with culling recommendations [33,39]. Expert surveys show 72.4% dissatisfaction with compensation conditions [34]. Importantly, while animal health control is a major determinant and cornerstone of human brucellosis prevention, it is not the sole factor; human incidence is also influenced by diagnostic delays, raw dairy consumption, occupational exposure, and reporting practices.
**Neglect of wildlife, nomadic lifestyles, and dogs:** *Brucella* infection has been reported in rodents and dogs, while brucellosis prevalence among nomadic populations has reached 6.3% and has been associated with high-risk practices, including raw-milk consumption [12,19,20,56]. Recent molecular surveys have substantially expanded the known wildlife host range: *B. abortus* was detected in two venomous snake species (*Pseudocerastes persicus* and *Vipera albicornuta*) at 2.2% prevalence [15], *Brucella* DNA was found in 0.68% of rodents (*Meriones persicus* and *Mus musculus*) in western Iran [17], and an outbreak on Kish Island confirmed *B. melitensis* in Persian goitered gazelles with 100% seroprevalence linked to infected livestock [18]. Despite these emerging data, Iran lacks a comprehensive national wildlife plan (PC06, score 1), which may increase the risk of reinfection in livestock populations [15].
**Communication as an afterthought:** Although training materials are available and KAP studies show moderate awareness [53,58], the absence of public annual reports (C06) and limited feedback to livestock owners (C05) may contribute to low risk perception and under‑reporting. The systematic review showing 22% contamination of dairy products highlights the urgent need for enhanced pasteurization promotion [6].

#### Recent post-search publications.

Since completing the systematic search in March 2026, several large-scale epidemiological studies have been published that corroborate concerns identified in our analysis across different production systems and geographic settings. A nationwide active surveillance program in industrial dairy cattle herds vaccinated with the RB51 vaccine (2021–2024) encompassed 33,994 herds and over 6.2 million blood samples across 32 provinces [66]. While farm-level prevalence declined from 9.7% in 2022 to 6.7% in 2024, the study confirmed the persistence of multiple *Brucella* biovars, including *B. abortus* biovars 1 and 3, *B. melitensis* biovars 1–3, and the vaccine strain RB51 [66]. The predominance of *B. melitensis* biovar 1, particularly in Qom and Alborz, raises concern about cross-species transmission from small ruminants to cattle and the ability of RB51 vaccination to address *B. melitensis* exposure. This concern directly aligns with our identification of inadequate vaccination coverage (PC-02, score 2) and the need for refined vaccination strategies. Concurrently, a cross-sectional study of small ruminant production systems (2021–2024) involving 389,225 animals from 3,929 herds reported low vaccination coverage (8–19.1%) across rural, traditional, and nomadic systems, with traditional farms having 3.4 times higher odds of animal-level brucellosis [67]. The reported association between increasing vaccination coverage and reduced infection odds (OR = 0.94 per 1% increase in coverage) is consistent with concerns regarding the adequacy of current vaccination coverage.

Furthermore, a spatio-temporal analysis of human brucellosis in North Khorasan (2015–2023) identified 2,855 confirmed cases, with 71.14% occurring in rural residents and 86.67% reporting consumption of unpasteurized dairy products [68]. Persistent high-incidence clusters in Bojnord, Shirvan, and Faruj from 2016 to 2023 provide additional evidence of a geographically concentrated burden in North Khorasan. These post-search publications were not used for indicator scoring but provide contextual corroboration of the persistence of brucellosis across multiple production systems, inadequate vaccination coverage in some populations, the disproportionate burden on rural and nomadic communities, and the need for region-specific and integrated control strategies—all of which are central to the six priority actions we propose.

### One Health integration remains weak

Our framework explicitly incorporated joint indicators (e.g., S04: data sharing; C04: joint risk communication; G09: joint risk assessment). The findings demonstrate that while individual sectoral capacities are moderate, the human–animal interface remains underdeveloped. No publicly accessible evidence of a formal joint risk-assessment protocol was identified (G09, score 0), and joint risk communication following outbreaks is infrequent (C04, score 1). Without routine joint risk assessment and data exchange, livestock-origin outbreaks may be recognized only after human cases emerge, missing opportunities for timely intervention. This underscores the necessity of the One Health approach advocated by WHO, FAO, and WOAH [14,28], and suggests that progression beyond Stage 3 would require both technical improvement and greater cross-sectoral integration. Previous assessments have often treated human brucellosis surveillance and livestock surveillance as separate exercises [40]. This study explicitly integrates human and animal health indicators across all five domains. Gaps in joint risk assessment (G09), data sharing (S04), and joint risk communication (C04) may delay recognition of livestock-origin outbreaks until human cases appear, limiting timely intervention and sustained reduction of human exposure.

### Six priority actions as a roadmap

Based on the gap analysis, we identified six interdependent evidence-informed priority actions linked to critical gaps and operationally incomplete enabling functions. We selected them based on each gap's documented severity and policy relevance, its potential program implications, and the feasibility of implementation within the existing Iranian institutional framework. No formal multi-criteria ranking was performed:

1. **Nationwide animal identification and traceability (G08)** — prerequisite for movement control; essential for rural and nomadic systems where animal identification is currently inadequate [1].
2. **Compensation reform (G04)** — faster payments and insurance linkage; critical for farmer compliance, as evidenced by 72.4% dissatisfaction with current compensation [34].
3. **Early warning system (S06)** — using syndromic abortion data and molecular typing; could help address the 4–6-month diagnostic delays observed in some provinces [40].
4. **Formal laboratory twinning (L05)** — external quality assurance and technical collaboration with a WOAH/FAO reference laboratory to support WOAH-compliant diagnostic capacity, including CFT [22].
5. **Institutionalized One Health governance (G01, G09) —** legally mandated joint risk assessment, as recommended by the Tripartite One Health framework [14,28]. Institutionalized One Health governance and public reporting could support earlier detection, improved occupational and food-safety risk reduction, and more effective monitoring of control outcomes across sectors.
6. **Sustained national awareness campaign with annual public reporting (C02, C06)** — must address nomadic lifestyle risks (94.7% raw-milk consumption and 100% animal-skin contact) [12] and promote pasteurization to address 22% dairy contamination [6].

These gaps could affect human health (continued exposure through raw dairy and occupational contact), animal health (persistent livestock reservoirs and the risk of reinfection), and cross-sectoral prevention (missed opportunities for coordinated early response).

These actions are interdependent. For example, early warning (priority 3) depends on laboratory confirmation (priority 4), and both require governance integration (priority 5). Collectively, they form a practical roadmap from Stage 3 to Stages 4 and 5. The six priority actions and the indicator framework can inform national planning, monitor progress through the SAPCB stages, and prioritize resources; prospective and external validation is required before broader application.

These priorities remain relevant despite the recent launch of a 5‑year mass vaccination campaign (Iranian Veterinary Organization press briefing, December 2024), as the historical gaps identified in this study—particularly in test‑and‑slaughter for small ruminants, wildlife/nomadic management, and intersectoral coordination—require sustained policy attention beyond vaccination alone.

### Limitations

Some limitations should be acknowledged. First, this is a policy gap analysis based on published literature and publicly available official documents rather than primary data collection. The evidence base may be incomplete or uneven and may not capture unpublished, confidential, or locally held information. For some indicators (e.g., S10, median abortion-to-confirmation time), a score of 0 indicates the absence of publicly available data. We distinguish “no publicly available evidence identified” from definitive evidence of absence and interpret such scores as evidence limitations rather than proof that the corresponding activity did not occur. Second, the authors developed and modified the 45-indicator framework from the FAO SAPCB and related international standards, which three subject-matter experts then content-reviewed. It has not been formally externally or prospectively validated. Therefore, residual subjectivity in the selection, wording, and scoring of indicators cannot be ruled out. The framework will require prospective and external validation before it can be used more widely outside the context of brucellosis control in Iran. Third, scoring was based on available documents and reviewers’ judgment. Although inter-rater reliability was calculated using the original independent pre-consensus scores, resolving six disagreements through consensus and subsequently reassessing four indicators after additional corroborating evidence was identified involved reviewer judgment and may have influenced the final indicator scores. Fourth, the framework emphasizes policy and systems rather than biological factors (e.g., strain diversity or vaccine efficacy) that may influence program outcomes. Fifth, excluding Embase and CAB Abstracts may have introduced selection bias. Including Scopus, Web of Science, and manual screening of reference lists and official documents likely reduced the risk of such bias. Lastly, the review protocol was not prospectively registered, which is recognized as a methodological limitation.

## Conclusions

Based on the adapted indicator framework and available documentary evidence, Iran met the minimum documented prerequisites for SAPCB Stage 3, while several operational functions required for advanced control and elimination remained incomplete or undocumented. This classification represents a framework-based interpretation rather than an official SAPCB designation or an independently validated measurement of national program performance. Stage classification and indicator scoring served distinct purposes: the stage reflects whether specified program-level prerequisites were documented, whereas individual scores describe the operational status of specific policies, systems, and capacities. Low or zero scores for individual indicators can therefore coexist with a Stage 3 classification.

Over 60 years of program implementation, coverage of vaccination has rarely reached the 80% threshold, and test-and-slaughter has covered less than 1% of the sheep and goat population [1]. The six evidence‑informed priority actions identified here provide a practical roadmap for overcoming these barriers. The framework offers a potentially transferable approach to systematic gap identification, but prospective and external validation is required before applying it in other endemic settings.

## Supporting information

S1 Appendix Supplementary materials for the scoping review. This file contains Table A (search syntax for PubMed, Scopus, Web of Science, and Google Scholar); Table B (the final 45-indicator matrix); Table C (the complete reference list of 71 included documents); Tables D–H (indicator-level scoring justifications for Governance, Surveillance, Laboratory, Prevention and Control, and Communication); Table I (the rule-based SAPCB stage-classification summary for Iran); Table J (the documentary evidence audit for indicators scoring 0 or 1); File A (the PRISMA-ScR checklist); and Text A (supplementary interpretation notes). The checklist items in File A in S1 Appendix are reproduced from Tricco AC et al. PRISMA *Extension for Scoping Reviews (*PRISMA*-ScR): Checklist and Explanation.* Ann Intern Med. 2018;169(7):467–473. https://doi.org/10.7326/M18-0850. Source checklist: https://www.prisma-statement.org/scoping. The manuscript-location entries were completed by the authors. Licensed under CC BY 4.0: https://creativecommons.org/licenses/by/4.0/.
(XLSX)
