# Supplementary material for: A one health indicator-based policy and implementation gap analysis of brucellosis control in Iran: A scoping review
Source: PLoS Negl Trop Dis. 2026 Nov 6;20(11):e0014690. doi: 10.1371/journal.pntd.0014690 (PMC13742969; doi:10.1371/journal.pntd.0014690)
Supplement: Supplementary file 1 [file pntd.0014690.s003.docx]

# Organized Reviewer Comments

**Manuscript ID:** PNTD-D-26-01569

**Title:** A One Health Indicator-Based Policy and Implementation Gap Analysis of Brucellosis Control in Iran: A Scoping Review

We thank the Editor and Reviewer for their constructive assessment. The revisions focused on clarifying the methods, improving internal consistency, and increasing reporting transparency. We updated the Supplementary Materials to align the indicator-stage crosswalk and to clarify the presentation of the scoring criteria, evidence justifications, stage-classification summary, evidence audit, and reporting checklist in accordance with the revised manuscript. These updates did not change the 45 final indicator scores, the inter-rater reliability results, the 71-document evidence corpus, the overall Stage 3 classification, or the principal conclusions.

## 1. Methods-related Comments

### 1.1 Objectives and hypotheses

The objectives are clearly articulated. The study aims to develop and apply an indicator-based framework to assess Iran's brucellosis control program and identify policy and implementation gaps. However, no explicit testable hypothesis is stated. Given that this is a scoping review and policy-gap analysis rather than an analytical epidemiological study, a formal hypothesis is not necessarily required. The authors should nevertheless clarify that the study is descriptive and exploratory and was not designed for conventional hypothesis testing.

**Response**

Thank you for this important comment. We agree. We have revised the Abstract, Introduction, and Methods to clarify that this study is a descriptive and exploratory scoping review combined with an indicator-based policy and implementation gap analysis. It was not designed to test a conventional epidemiological hypothesis or estimate causal effects. We have also clarified that conventional sample-size and statistical-power calculations are not applicable because the unit of analysis is a corpus of published literature and publicly accessible official documents rather than a human or animal study population.

**Revised manuscript text:**

**Abstract (Background**): "We conducted a descriptive and exploratory scoping review combined with an indicator-based policy and implementation gap analysis. The study was not designed to test a conventional epidemiological hypothesis or estimate causal effects."

**Introduction (final paragraph):** "To address this gap, we conducted a descriptive and exploratory scoping review combined with an indicator-based policy and implementation gap analysis. The study was not designed to test a conventional epidemiological hypothesis or estimate causal effects."

**Methods (opening paragraph):** "This study is a descriptive and exploratory scoping review combined with an indicator-based policy and implementation gap analysis. It was not designed to test a conventional epidemiological hypothesis, estimate causal effects, or generate population-level effect estimates. Conventional sample-size and statistical-power calculations were therefore not applicable because the unit of analysis was the document corpus rather than a human or animal study population."

### 1.2 Appropriateness of the study design

Yes. The combination of a scoping review with an indicator-based policy and implementation gap analysis is appropriate for the stated objectives. The use of predefined indicators, document screening, independent scoring by two reviewers, consensus resolution, and expert review provides a reasonable methodological framework. However, the manuscript would benefit from a clearer explanation and justification of how the 45 indicators were developed, adapted, and validated.

**Response**

Thank you for this constructive comment. We have expanded the Methods section (Phase I — Indicator development) to provide a more transparent description of the process. Specifically, we now explain that we generated the indicators by systematically translating the FAO SAPCB stage descriptors and key activities into measurable, closed-ended questions across the five domains (Governance, Surveillance, Laboratory, Prevention & Control, and Communication). We cross-checked candidate indicators against the nine normative sources listed in Table 2. We consolidated overlapping indicators and removed those that could not be assessed from publicly available Iranian scientific, official, or grey literature.

We also clarified the role of the three subject-matter experts, who independently evaluated every indicator against three criteria: (i) relevance to the burden and control landscape of brucellosis in Iran, (ii) clarity and measurability, and (iii) feasibility of data retrieval from Iranian sources. Based on their feedback, we refined the wording and scope of the indicators, after which all three experts agreed on the final set of 45 indicators.

We have explicitly stated that this process constituted conceptual adaptation and content review rather than formal psychometric, prospective, or external validation. The framework was developed and adapted specifically for this study and requires prospective and external validation before broader application. Corresponding clarifications have been added to the Limitations section.

**Location of change:** Methods (Phase I — Indicator development); Limitations section.

### 1.3 Study population/evidence base

Not applicable in the conventional sense. This study does not involve a human or animal study population. The analysis is based on published literature and publicly accessible official documents concerning brucellosis control in Iran. The eligibility criteria and evidence sources are described. The authors should therefore refer to the included evidence/document corpus rather than a study population and clarify the limitations associated with the completeness and representativeness of the available documentary evidence.

**Response**

Thank you for this comment. We agree that the manuscript should consistently describe the included sources as an evidence base or document corpus rather than a study population, and should clarify the associated limitations.

We have revised the Methods and Limitations sections to replace any reference to a "study population" with "document corpus," "evidence base," or "included sources." We now state that the analysis is based on a corpus of 71 documents identified through the stated eligibility criteria, and that the absence of accessible documentary evidence should not be interpreted as definitive proof that an activity did not occur. The limitations associated with the completeness and representativeness of publicly available documents have also been expanded. To make this distinction transparent at the indicator level, Supplementary File S6 now audits the documentary basis for all 26 indicators scoring 0 or 1 and explicitly states that failure to identify publicly accessible evidence should not be interpreted as definitive proof that an activity did not occur.

**Location of change:** Methods (Eligibility criteria; Screening and data extraction); Discussion (Limitations); Supplementary File S6.

### 1.4 Sample size and statistical power

Not applicable. This is not a hypothesis-testing study, and therefore a conventional sample-size or statistical power calculation is not required. The review included 71 key documents after the screening process. The authors should explicitly state that statistical power calculations are not applicable to this type of scoping and policy-analysis study.

**Response**

Thank you for this comment. We agree that the manuscript should explicitly state that conventional sample-size and statistical-power calculations are not applicable.

We added a clear statement in the Methods section clarifying that this is a descriptive, exploratory scoping review and policy-gap analysis, not a hypothesis-testing epidemiological study, and that power calculations are therefore not required. The unit of analysis is the document corpus (71 documents), not a human or animal study population.

**Location of change:** Methods (Study design and analytical framework / Screening and data extraction).

### 1.5 Statistical analysis and inter-rater reliability

Generally yes. The study primarily uses descriptive indicator scoring rather than inferential statistical testing. The use of weighted Cohen's kappa to assess agreement between reviewers is appropriate for ordinal scoring. However, the manuscript indicates that the final scores were obtained after discrepancies were resolved by consensus. Reliability should ideally be calculated using the independent pre-consensus scores, because calculating agreement after consensus may overestimate inter-rater reliability. The authors should clarify this point and, if possible, report the pre-consensus agreement statistics. The rationale for converting indicator scores into overall SAPCB stage classifications should also be described more transparently.

**Response**

Thank you for this important methodological comment. We agree that inter-rater reliability should be assessed using the reviewers' original independent scores rather than consensus-resolved scores. After reviewing the original scoring records, we confirmed that we calculated weighted Cohen’s kappa with quadratic weights using the original independent pre-consensus scores. The previous manuscript version did not clearly describe the input scores used for this calculation, and we have now corrected this description.

Exact agreement was observed for 39 of the 45 indicators (86.7%). For the remaining six indicators, the two independent scores differed by one category on the 0–3 ordinal scale. Weighted Cohen's kappa was 0.84 (95% CI 0.72–0.93). The six disagreements were subsequently resolved through discussion and consensus after both reviewers re-examined the supporting evidence.

We have also clarified that, following the initial scoring and consensus process, an independent third reviewer identified additional corroborating evidence relevant to four indicators. The two original reviewers reassessed these indicators in light of the additional evidence and agreed on the final scores. We did not include these later evidence-based revisions in the inter-rater reliability calculation.

We also clarified how the stage-classification algorithm treated performance thresholds. For S-01, we used the 10-year recency criterion as the benchmark for indicator score 3, while the stage prerequisite was the documented presence of a national seroprevalence survey. Similarly, for PC-02, we used the ≥80% vaccination-coverage threshold as the benchmark for indicator score 3, while documented routine vaccination in high-risk areas was the minimum prerequisite for Stage 3 classification. These clarifications did not change either indicator score or the overall stage classification.

Finally, we clarified that individual indicator scores were not combined into a composite score and were not used to determine the overall SAPCB stage. We conducted overall stage classification separately using the rule-based algorithm based on documented program prerequisites described in the Methods.

**Location of change:** Methods (Scoring and gap analysis; Stage classification algorithm); Discussion (Limitations); Supplementary Table S2; Supplementary Files S4 and S5.

### 1.6 Ethical and regulatory requirements

No major ethical or regulatory concerns are apparent. The study used published literature and publicly accessible official documents and did not involve primary data collection from humans or animals. The authors appropriately state that ethics approval was therefore not required. The manuscript also indicates that the review protocol was not prospectively registered, which should be acknowledged as a methodological limitation rather than an ethical concern.

**Response**

Thank you for this comment. We have retained the statement that ethics approval was not required because the study used only published literature and publicly accessible official documents and involved no primary data collection from humans or animals. We clarified in the Limitations section that the absence of prospective protocol registration is a methodological limitation.

**Location of change:** Methods (Ethical statement); Discussion (Limitations).

## 2. Results-related Comments

### 2.1 Consistency with the analytical plan

Generally yes. The analyses presented are consistent with the stated objectives and methodology. The study uses descriptive scoring of the 45 indicators and inter-rater agreement analysis, which corresponds to the planned indicator-based assessment. However, the manuscript should provide a clearer description of the analytical workflow, particularly how individual indicator scores were aggregated and how the final SAPCB stage classification was derived. The calculation of inter-rater reliability should also be clarified, especially whether Cohen's kappa was calculated from the independent scores before consensus was reached.

**Response**

Thank you for this comment. We have clarified the analytical workflow in the revised Methods. Each of the 45 indicators was independently scored on a 0–3 scale using predefined criteria. We did not combine the scores into a composite score or use them to determine the overall SAPCB stage. We calculated domain-by-stage means solely for descriptive visualization in Figure 2. We conducted overall stage classification separately using a rule-based algorithm based on documented program prerequisites.

We confirmed that we calculated weighted Cohen’s kappa using the original independent pre-consensus scores, and we now report the subsequent consensus process and third-reviewer evidence reassessment as separate steps. We further clarified that the recency and coverage thresholds used to distinguish fully operational indicator performance were not treated as independent stage-attainment thresholds.

**Location of change:** Location of change: Methods (Scoring and gap analysis; Stage classification algorithm); Figure 2 legend; Supplementary Table S2; Supplementary File S5.

### 2.2 Completeness and clarity of results

The results are generally clearly presented and address the main objectives of the study. The progression from document screening to indicator assessment and identification of policy and implementation gaps is understandable. The identification of the six priority actions is also useful. However, some methodological details underlying the final scores and stage classification should be presented more transparently so that readers can reproduce or independently assess the conclusions. It would also be helpful to distinguish more clearly between evidence directly supported by the reviewed documents and interpretations derived from the authors' indicator framework.

**Response**

Thank you for this comment. We clarified in the Methods and Results that indicator scores reflect application of the predefined scoring criteria to the available documentary evidence, whereas the SAPCB stage classification is a framework-based interpretation derived separately from the rule-based algorithm. We now distinguish more explicitly between information reported directly in the reviewed documents and interpretations generated by applying the study-specific framework.

We revised the Supplementary Materials to make this analytical distinction independently assessable. Supplementary Table S2 presents the final indicator matrix, stage assignments, normative sources, and scoring criteria. Supplementary File S4 provides indicator-level supporting sources and final scoring justifications. Supplementary File S5 reports the documentary basis for each stage-classification decision. Supplementary File S6 audits the evidence supporting every indicator scoring 0 or 1 and distinguishes absence of publicly accessible evidence from definitive evidence of absence.

We also revised Table 8 so that each selected indicator is presented in a separate row and assigned an indicator-specific primary gap type. This presentation change improves interpretability and avoids applying a single gap type to multiple indicators with different documentary profiles. It did not change any indicator score or policy priority.

**Location of change:** Methods (Scoring and gap analysis; Stage classification algorithm); Results (Analytical framework and 45 indicators; Critical gaps; Table 8); Supplementary Table S2; Supplementary Files S4–S6.

### 2.3 Quality of figures and tables

The tables and figures are generally adequate for presenting the study findings and indicator-based assessment. The presentation of the 45 indicators and their corresponding scores is particularly important and should remain sufficiently detailed to allow readers to understand how the conclusions were reached. The authors should ensure that all tables and figures have clear titles, explanatory legends, defined abbreviations, and readable text. If any figure presents the SAPCB stages or indicator results in a highly condensed format, additional explanation may be needed to make the classification and comparison readily understandable.

**Response**

Thank you for this comment. We reviewed the tables and figures to improve the clarity of their titles, legends, column headings, and explanatory notes. To resolve ambiguity between indicator placement in the matrix and the documentary prerequisites specified in the rule-based algorithm, we aligned the indicator-stage crosswalk across the manuscript, Figure 2, and the Supplementary Materials. G-03, PC-02, L-01, and L-04 are displayed as Stage 3 indicators; S-05 and PC-05 as Stage 4 transition indicators; and G-06, S-07, L-06, PC-07, and C-06 as Stage 5 indicators.

Domain–stage cells without an assigned indicator are shown as not applicable (N/A) and are excluded from mean calculations. The revised Figure 2 legend explains that the heatmap means are descriptive and were not used to determine the overall country-level stage classification.

Table 8 was also reformatted to present one indicator per row, with the primary gap type assigned individually based on the dominant documentary limitation. These revisions changed indicator-stage presentation and the corresponding descriptive heatmap means but did not change any indicator score, the overall Stage 3 classification, or the six policy priorities.

**Location of change:** Figure 2 and its legend; Tables 3–8; Supplementary Table S2; Supplementary Files S4–S6.

## 3. Conclusions-related Comments

### 3.1 Support for the conclusions

Generally yes. The main conclusions are supported by the indicator-based assessment and the reviewed documentary evidence. The conclusion that Iran meets prerequisites associated with SAPCB Stage 3, while important operational indicators for Stages 3–5 remain largely unachieved, is consistent with the results presented. However, because the 45-indicator framework was developed and adapted by the authors, the conclusions should be presented as an assessment based on this framework rather than as an objective measurement of national program performance. The authors should also avoid implying causality where the available evidence only demonstrates policy or implementation gaps.

**Response**

Thank you for this comment. We agree. We have revised the wording in the Abstract, Results, Discussion, and Conclusions to present the SAPCB Stage 3 classification as an assessment based on the adapted indicator framework and the reviewed documents. We have also revised language that could imply causality, replacing it with formulations that describe associations or potential constraints.

**Location of change:** Abstract (Methodology/Principal Findings and Conclusions/Significance); Results (Analytical framework paragraph); Discussion (opening and stage-related paragraphs); Conclusions.

### 3.2 Limitations

The manuscript identifies several important limitations, including the absence of prospective registration and limitations associated with the available evidence. However, the limitations could be discussed more comprehensively. In particular, the authors should acknowledge the potential subjectivity involved in developing and scoring the 45 indicators, the limited external validation of the framework, possible incompleteness or uneven availability of national documents, and the possibility that consensus-based scoring may influence the assessment. The generalizability of the findings beyond the Iranian brucellosis control context should also be addressed.

**Response**

Thank you for this comment. We agree that the limitations should be discussed more comprehensively. We expanded the Limitations section to acknowledge: (1) potential subjectivity in the development, interpretation, and scoring of the 45 indicators; (2) the absence of formal prospective, external, or psychometric validation of the adapted framework; (3) possible incompleteness and uneven public availability of national documents; (4) the potential influence of reviewer judgment during consensus resolution and the subsequent evidence-based reassessment of four indicators on the final scores; (5) limited generalizability beyond the Iranian brucellosis-control context; and (6) the absence of prospective protocol registration.

We also clarified that the weighted kappa was calculated using the original independent pre-consensus scores. The consensus process influenced the final indicator scores but did not form the basis of the inter-rater reliability calculation.

**Location of change:** Discussion (Limitations).

**3.3 Contribution to understanding and future application**

Yes. The manuscript demonstrates how an indicator-based framework can be used to systematically identify gaps between policy requirements and implementation of brucellosis control measures. The identification of specific priority actions provides a practical basis for strengthening surveillance, laboratory capacity, prevention and control, governance, and communication. The potential value of the framework for monitoring progress through the SAPCB stages should be emphasized, while recognizing that prospective validation would be necessary before broader application.

**Response**

Thank you for this comment. We have added a short statement in the Discussion and Conclusions clarifying that the indicator framework and the six priority actions can support national planning, monitoring of progress across SAPCB stages, and resource prioritization, and that prospective and external validation is required before wider use.

**Location of change:** Discussion (Six priority actions as a roadmap / closing paragraph); Conclusions.

### 3.4 Public health relevance

Yes. Public health relevance is clearly inherent in the study, as brucellosis is a zoonotic disease and effective control requires coordinated actions across human, animal, and related sectors. The findings identify gaps that may affect the effectiveness of national brucellosis prevention and control efforts. The One Health perspective and the identified priority actions are therefore relevant to public health planning and disease control. The discussion could be strengthened by explicitly linking each major identified gap to its potential implications for human health, animal health, and cross-sectoral disease prevention.

**Response**

Thank you for this comment. We have expanded the Discussion (particularly the One Health integration subsection and the six priority actions) to connect the key gaps—limited joint risk assessment and data sharing, incomplete animal identification, delayed compensation, lack of documented early-warning capacity, absence of formal laboratory twinning, and weak risk communication—with their potential implications for delayed detection of livestock outbreaks, continued human exposure through raw dairy and occupational contact, and reduced effectiveness of coordinated prevention.

**Location of change:** Discussion (One Health integration remains weak; Six priority actions as a roadmap).

## 4. Summary and General Comments (Reviewer #1)

The manuscript has a generally appropriate design for a scoping/policy-gap analysis, but the authors should clarify that it is not a hypothesis-testing study. The principal methodological concern is the construction and validation of the 45-indicator framework and, particularly, the calculation of inter-rater reliability after consensus. The manuscript itself acknowledges that the post-consensus κ may overestimate the true pre-consensus agreement. I would therefore recommend minor to moderate methodological clarification, rather than questioning the overall study design.

**Response**

Thank you for this balanced and constructive overall assessment. In response, we clarified throughout the Abstract, Introduction, and Methods that the study is descriptive and exploratory and was not designed for conventional epidemiological hypothesis testing or causal inference. We also expanded the description of how we developed and adapted the 45-indicator framework and clarified that the expert-review process constituted conceptual adaptation and content review rather than formal psychometric, prospective, or external validation.

Upon reviewing the original scoring records, we confirmed that weighted Cohen’s kappa had been calculated using the original independent pre-consensus scores. The previous manuscript version did not clearly describe the input scores used for this calculation, and we have corrected this description. Exact agreement was observed for 39 of the 45 indicators (86.7%), and the remaining six scores differed by one category. Weighted Cohen's kappa was 0.84 (95% CI 0.72–0.93). We now report the subsequent consensus process and later evidence-based reassessment of four indicators separately from the inter-rater reliability analysis.

We believe these revisions provide the requested minor-to-moderate methodological clarifications and improve the transparency and reproducibility of the work without altering the overall study design, indicator scores, or principal conclusions.

We revised the Supplementary Materials in parallel solely to improve documentation and cross-file consistency. S1 clarifies the reconstructed search syntax; S2 presents the final indicator matrix and scoring criteria; S3 confirms the 71-document evidence corpus; S4 provides indicator-level scoring justifications; S5 aligns the stage-classification summary with the rule-based algorithm; S6 provides an evidence audit for indicators scoring 0 or 1; and S7 updates the PRISMA-ScR reporting locations. These revisions did not introduce new indicators, change the evidence corpus, rescore any indicator, alter the inter-rater reliability results, or modify the overall Stage 3 conclusion.

**Location of change:** Abstract; Introduction (final paragraph); Methods (Study design and analytical framework; Phase I — Indicator development; Scoring and gap analysis; Stage classification algorithm); Results (Analytical framework and 45 indicators; Figure 2; Table 8); Discussion (Limitations); Conclusions; Supplementary Files S1–S7.

## 5. Journal Requirements (for reference)

1) Please ensure that the CRediT author contributions listed for every co-author are completed accurately and in full. At this stage, the following Authors/Authors require contributions: Zahra Baradaran-Seyed.

Complete the CRediT contributions for Zahra Baradaran-Seyed in the Editorial Manager

**Response**

The CRediT contributions for Zahra Baradaran-Seyed have been completed and verified in Editorial Manager and are consistent with the Author Contributions statement in the revised manuscript.

2) Please upload all main figures as separate Figure files in .tif or .eps format.

3) Please upload a copy of Figure Fig 1 and 2 which you refer to in your text on pages 8, and 9. Or, if the figure is no longer to be included as part of the submission please remove all reference to it within the text.

**Response**

Figure 1 and Figure 2 have been uploaded as separate main-figure files in TIFF format. All in-text references to these figures have been retained and checked against the revised manuscript.
