# Supplementary material for: A one health indicator-based policy and implementation gap analysis of brucellosis control in Iran: A scoping review
Source: PLoS Negl Trop Dis. 2026 Nov 6;20(11):e0014690. doi: 10.1371/journal.pntd.0014690 (PMC13742969; doi:10.1371/journal.pntd.0014690)
Supplement: Peer review history

# Peer review: Decision Letter 0

**DOI:** 10.1371/journal.pntd.0014690.r001

**Authors:** Ana LTO Nascimento, Lawrence Mugisha

7 Oct 2026

Response to Reviewers Revised Manuscript with Track Changes Manuscript

Shaden Kamhawi

co-Editor-in-Chief

PLOS Neglected Tropical Diseases

orcid.org/0000-0003-4304-636XX

Paul Brindley

co-Editor-in-Chief

PLOS Neglected Tropical Diseases

orcid.org/0000-0003-1765-0002

**Journal Requirements:**

1) Please ensure that the CRediT author contributions listed for every co-author are completed accurately and in full.

At this stage, the following Authors/Authors require contributions: Zahra Baradaran-Seyed. Please ensure that the full contributions of each author are acknowledged in the "Add/Edit/Remove Authors" section of our submission form.

The list of CRediT author contributions may be found here: https://journals.plos.org/plosntds/s/authorship#loc-author-contributions

2) Please upload all main figures as separate Figure files in .tif or .eps format. For more information about how to convert and format your figure files please see our guidelines:

https://journals.plos.org/plosntds/s/figures

3) Please upload a copy of Figure Fig 1 and 2 which you refer to in your text on pages 8, and 9. Or, if the figure is no longer to be included as part of the submission please remove all reference to it within the text.

**Reviewers' comments:**

**Key Review Criteria Required for Acceptance?**

As you describe the new analyses required for acceptance, please consider the following:

**Methods**

-Are the objectives of the study clearly articulated with a clear testable hypothesis stated?

-Is the study design appropriate to address the stated objectives?

-Is the population clearly described and appropriate for the hypothesis being tested?

-Is the sample size sufficient to ensure adequate power to address the hypothesis being tested?

-Were correct statistical analysis used to support conclusions?

-Are there concerns about ethical or regulatory requirements being met?

Reviewer #1: Are the objectives of the study clearly articulated with a clear testable hypothesis stated?

Yes, the objectives are clearly articulated. The study aims to develop and apply an indicator-based framework to assess Iran’s brucellosis control program and identify policy and implementation gaps. However, no explicit testable hypothesis is stated. Given that this is a scoping review and policy-gap analysis rather than an analytical epidemiological study, a formal hypothesis is not necessarily required. The authors should nevertheless clarify that the study is descriptive and exploratory and was not designed for conventional hypothesis testing.

Is the study design appropriate to address the stated objectives?

Yes. The combination of a scoping review with an indicator-based policy and implementation gap analysis is appropriate for the stated objectives. The use of predefined indicators, document screening, independent scoring by two reviewers, consensus resolution, and expert review provides a reasonable methodological framework. However, the manuscript would benefit from a clearer explanation and justification of how the 45 indicators were developed, adapted, and validated.

Is the population clearly described and appropriate for the hypothesis being tested?

Not applicable in the conventional sense. This study does not involve a human or animal study population. The analysis is based on published literature and publicly accessible official documents concerning brucellosis control in Iran. The eligibility criteria and evidence sources are described. The authors should therefore refer to the included evidence/document corpus rather than a study population and clarify the limitations associated with the completeness and representativeness of the available documentary evidence.

Is the sample size sufficient to ensure adequate power to address the hypothesis being tested?

Not applicable. This is not a hypothesis-testing study and therefore a conventional sample-size or statistical power calculation is not required. The review included 71 key documents after the screening process. The authors should explicitly state that statistical power calculations are not applicable to this type of scoping and policy-analysis study.

Were correct statistical analyses used to support conclusions?

Generally yes. The study primarily uses descriptive indicator scoring rather than inferential statistical testing. The use of weighted Cohen’s kappa to assess agreement between reviewers is appropriate for ordinal scoring. However, the manuscript indicates that the final scores were obtained after discrepancies were resolved by consensus. Reliability should ideally be calculated using the independent pre-consensus scores, because calculating agreement after consensus may overestimate inter-rater reliability. The authors should clarify this point and, if possible, report the pre-consensus agreement statistics. The rationale for converting indicator scores into overall SAPCB stage classifications should also be described more transparently.

Are there concerns about ethical or regulatory requirements being met?

No major ethical or regulatory concerns are apparent. The study used published literature and publicly accessible official documents and did not involve primary data collection from humans or animals. The authors appropriately state that ethics approval was therefore not required. The manuscript also indicates that the review protocol was not prospectively registered, which should be acknowledged as a methodological limitation rather than an ethical concern.

**********

**Results**

-Does the analysis presented match the analysis plan?

-Are the results clearly and completely presented?

-Are the figures (Tables, Images) of sufficient quality for clarity?

Reviewer #1: Does the analysis presented match the analysis plan?

Generally yes. The analyses presented are consistent with the stated objectives and methodology. The study uses descriptive scoring of the 45 indicators and inter-rater agreement analysis, which corresponds to the planned indicator-based assessment. However, the manuscript should provide a clearer description of the analytical workflow, particularly how individual indicator scores were aggregated and how the final SAPCB stage classification was derived. The calculation of inter-rater reliability should also be clarified, especially whether Cohen’s kappa was calculated from the independent scores before consensus was reached.

Are the results clearly and completely presented?

The results are generally clearly presented and address the main objectives of the study. The progression from document screening to indicator assessment and identification of policy and implementation gaps is understandable. The identification of the six priority actions is also useful. However, some methodological details underlying the final scores and stage classification should be presented more transparently so that readers can reproduce or independently assess the conclusions. It would also be helpful to distinguish more clearly between evidence directly supported by the reviewed documents and interpretations derived from the authors’ indicator framework.

Are the figures (Tables, Images) of sufficient quality for clarity?

The tables and figures are generally adequate for presenting the study findings and indicator-based assessment. The presentation of the 45 indicators and their corresponding scores is particularly important and should remain sufficiently detailed to allow readers to understand how the conclusions were reached. The authors should ensure that all tables and figures have clear titles, explanatory legends, defined abbreviations, and readable text. If any figure presents the SAPCB stages or indicator results in a highly condensed format, additional explanation may be needed to make the classification and comparison readily understandable.

**********

**Conclusions**

-Are the conclusions supported by the data presented?

-Are the limitations of analysis clearly described?

-Do the authors discuss how these data can be helpful to advance our understanding of the topic under study?

-Is public health relevance addressed?

Reviewer #1: Are the conclusions supported by the data presented?

Generally yes. The main conclusions are supported by the indicator-based assessment and the reviewed documentary evidence. The conclusion that Iran meets prerequisites associated with SAPCB Stage 3, while important operational indicators for Stages 3–5 remain largely unachieved, is consistent with the results presented. However, because the 45-indicator framework was developed and adapted by the authors, the conclusions should be presented as an assessment based on this framework rather than as an objective measurement of national program performance. The authors should also avoid implying causality where the available evidence only demonstrates policy or implementation gaps.

Are the limitations of analysis clearly described?

The manuscript identifies several important limitations, including the absence of prospective registration and limitations associated with the available evidence. However, the limitations could be discussed more comprehensively. In particular, the authors should acknowledge the potential subjectivity involved in developing and scoring the 45 indicators, the limited external validation of the framework, possible incompleteness or uneven availability of national documents, and the possibility that consensus-based scoring may influence the assessment. The generalizability of the findings beyond the Iranian brucellosis control context should also be addressed.

Do the authors discuss how these data can be helpful to advance our understanding of the topic under study?

Yes. The manuscript demonstrates how an indicator-based framework can be used to systematically identify gaps between policy requirements and implementation of brucellosis control measures. The identification of specific priority actions provides a practical basis for strengthening surveillance, laboratory capacity, prevention and control, governance, and communication. The potential value of the framework for monitoring progress through the SAPCB stages should be emphasized, while recognizing that prospective validation would be necessary before broader application.

Is public health relevance addressed?

Yes. Public health relevance is clearly inherent in the study, as brucellosis is a zoonotic disease and effective control requires coordinated actions across human, animal, and related sectors. The findings identify gaps that may affect the effectiveness of national brucellosis prevention and control efforts. The One Health perspective and the identified priority actions are therefore relevant to public health planning and disease control. The discussion could be strengthened by explicitly linking each major identified gap to its potential implications for human health, animal health, and cross-sectoral disease prevention.

**********

**Editorial and Data Presentation Modifications?**

Use this section for editorial suggestions as well as relatively minor modifications of existing data that would enhance clarity. If the only modifications needed are minor and/or editorial, you may wish to recommend “Minor Revision” or “Accept”.

Reviewer #1: 1. Clarify the study objective and analytical nature of the work. The authors should explicitly state that the study is a scoping and policy-gap analysis and is not designed to test a conventional epidemiological hypothesis.

2. Provide a more detailed justification for the development and selection of the 45 indicators. The manuscript should explain how the indicators were adapted from the FAO SAPCB framework, why each domain and indicator was considered relevant, and whether any indicators were modified or excluded during development.

3. Clarify the scoring methodology. The authors should describe precisely how individual indicators were scored, how scores were aggregated across domains, and how the overall SAPCB stage was assigned. The criteria for classifying Iran as meeting the prerequisites for Stage 3 should be explicitly presented.

4. Clarify inter-rater reliability analysis. Cohen’s kappa should preferably be calculated using the two reviewers’ independent scores before consensus resolution. If the reported kappa was calculated after consensus, this should be corrected or clearly acknowledged because consensus-based scoring can inflate apparent agreement.

5. Strengthen the description of the evidence base. Since there was no human or animal study population, the manuscript should consistently describe the included literature and official documents as the evidence base or document corpus rather than referring to a study population.

6. Expand the limitations section. In addition to the limitations already identified, the authors should discuss the potential subjectivity of indicator development and scoring, limited external validation of the 45-indicator framework, possible incompleteness of publicly available documents, differences in the quality of documentary evidence, and limitations in generalizing the framework beyond the Iranian context.

7. Distinguish evidence from interpretation more explicitly. The manuscript should clearly differentiate findings directly supported by reviewed documents from conclusions generated through application of the authors’ indicator framework.

8. Moderate conclusions where appropriate. Statements concerning Iran’s SAPCB stage should be framed as an assessment according to the proposed indicator framework rather than as an independently validated measurement of national program performance.

9. Strengthen the discussion of practical implications. The authors should explain how the six identified priority actions could inform national brucellosis control planning, monitoring, resource allocation, and progression through the SAPCB stages.

10. Strengthen the One Health and public health implications. The discussion should explicitly connect the identified gaps with potential implications.

**********

**Summary and General Comments**

Use this section to provide overall comments, discuss strengths/weaknesses of the study, novelty, significance, general execution and scholarship. You may also include additional comments for the author, including concerns about dual publication, research ethics, or publication ethics. If requesting major revision, please articulate the new experiments that are needed.

Reviewer #1: The manuscript has a generally appropriate design for a scoping/policy-gap analysis, but the authors should clarify that it is not a hypothesis-testing study. The principal methodological concern is the construction and validation of the 45-indicator framework and, particularly, the calculation of inter-rater reliability after consensus. The manuscript itself acknowledges that the post-consensus κ may overestimate the true pre-consensus agreement.

I would therefore recommend minor to moderate methodological clarification, rather than questioning the overall study design.

**********

PLOS authors have the option to publish the peer review history of their article (what does this mean?). If published, this will include your full peer review and any attached files.

If you choose “no”, your identity will remain anonymous but your review may still be made public.

**Do you want your identity to be public for this peer review?**  For information about this choice, including consent withdrawal, please see our Privacy Policy.

Reviewer #1: **Yes:**  Anton Gerilovych

**Figure resubmission:**

**Reproducibility:** -->To enhance the reproducibility of your results, we recommend that authors of applicable studies deposit laboratory protocols in protocols.io, where a protocol can be assigned its own identifier (DOI) such that it can be cited independently in the future. Additionally, PLOS ONE offers an option to publish peer-reviewed clinical study protocols. Read more information on sharing protocols at https://plos.org/protocols?utm_medium=editorial-email&utm_source=authorletters&utm_campaign=protocols-->?>

# Author comment: Author response to Decision Letter 1

**DOI:** 10.1371/journal.pntd.0014690.r002

13 Oct 2026

Attachment Submitted filename: Response to Reviewer Comments_PNTD-D-26-01569.docx

# Editor report: Decision Letter 1

**DOI:** 10.1371/journal.pntd.0014690.r003

**Authors:** Ana LTO Nascimento, Lawrence Mugisha

20 Oct 2026

Dear Dr. Baradaran-Seyed,

We are pleased to inform you that your manuscript 'A One Health Indicator-Based Policy and Implementation Gap Analysis of Brucellosis Control in Iran: A Scoping Review' has been provisionally accepted for publication in PLOS Neglected Tropical Diseases.

Before your manuscript can be formally accepted you will need to complete some formatting changes, which you will receive in a follow up email. A member of our team will be in touch with a set of requests.

Please note that your manuscript will not be scheduled for publication until you have made the required changes, so a swift response is appreciated.

IMPORTANT: The editorial review process is now complete. PLOS will only permit corrections to spelling, formatting or significant scientific errors from this point onwards. Requests for major changes, or any which affect the scientific understanding of your work, will cause delays to the publication date of your manuscript.

Should you, your institution's press office or the journal office choose to press release your paper, you will automatically be opted out of early publication. We ask that you notify us now if you or your institution is planning to press release the article. All press must be co-ordinated with PLOS.

Thank you again for supporting Open Access publishing; we are looking forward to publishing your work in PLOS Neglected Tropical Diseases.

Best regards,

Lawrence Mugisha, PhD

Academic Editor

PLOS Neglected Tropical Diseases

Ana LTO Nascimento

Section Editor

PLOS Neglected Tropical Diseases

Shaden Kamhawi

co-Editor-in-Chief

PLOS Neglected Tropical Diseases

orcid.org/0000-0003-4304-636XX

Paul Brindley

co-Editor-in-Chief

PLOS Neglected Tropical Diseases

orcid.org/0000-0003-1765-0002

***********************************************************

# Editor report: Acceptance letter

**DOI:** 10.1371/journal.pntd.0014690.r004

**Authors:** Ana LTO Nascimento, Lawrence Mugisha

Dear Dr Baradaran-Seyed,

We are delighted to inform you that your manuscript, "A One Health Indicator-Based Policy and Implementation Gap Analysis of Brucellosis Control in Iran: A Scoping Review," has been formally accepted for publication in PLOS Neglected Tropical Diseases.

We have now passed your article onto the PLOS Production Department who will complete the rest of the publication process. All authors will receive a confirmation email upon publication.

The corresponding author will soon be receiving a typeset proof for review, to ensure errors have not been introduced during production. Please review the PDF proof of your manuscript carefully, as this is the last chance to correct any scientific or type-setting errors. Please note that major changes, or those which affect the scientific understanding of the work, will likely cause delays to the publication date of your manuscript. Note: Proofs for Front Matter articles (Editorial, Viewpoint, Symposium, Review, etc...) are generated on a different schedule and may not be made available as quickly.

Soon after your final files are uploaded, the early version of your manuscript will be published online unless you opted out of this process. The date of the early version will be your article's publication date. The final article will be published to the same URL, and all versions of the paper will be accessible to readers.

For Research Articles, you will receive an invoice from PLOS for your publication fee after your manuscript has reached the completed accept phase. If you receive an email requesting payment before acceptance or for any other service, this may be a phishing scheme. Learn how to identify phishing emails and protect your accounts at https://explore.plos.org/phishing.

Thank you again for supporting open-access publishing; we are looking forward to publishing your work in PLOS Neglected Tropical Diseases.

Best regards,

Shaden Kamhawi

co-Editor-in-Chief

PLOS Neglected Tropical Diseases

Paul Brindley

co-Editor-in-Chief

PLOS Neglected Tropical Diseases
